# Stigma and depression among patients with tuberculosis in Khyber Pakhtunkhwa Pakistan: a cross-sectional study

**DOI:** 10.1186/s12889-026-27655-z

**Published:** 2026-05-23

**Authors:** Bushra Bibi, Zeeshan Kibria, Rubab Jawed, Khalid Rehman, Mark Goodall, Mirrat Gul, Ghasem Yadegarfar

**Affiliations:** 1 Office of the Research Innovation & Commercialization, Khyber Medical University, Peshawar, Pakistan; 2 Institute of Public Health and Social Sciences, Khyber Medical University, Peshawar, Pakistan; 3 Primary Care and Mental Health, Institute of Population Health, University of Liverpool, Liverpool, UK; 4 Mayo Hospital, Lahore, Pakistan; 5 Keele University, Newcastle-under-Lyme, UK

**Keywords:** Depression, Prevalence, Mental Health, Patient Perspective Stigma, Community Perspective Stigma, Tuberculosis, Pakistan

## Abstract

**Background:**

Tuberculosis (TB), a highly stigmatized disease, remains a major public health challenge globally, with stigma and mental health issues such as depression significantly impacting patient outcomes. TB-related stigma undermines patient engagement with testing and treatment, driven by factors at the individual, community, and health-system levels. In Pakistan, particularly in the Khyber Pakhtunkhwa region, the interplay between TB-related stigma and depression is underexplored. Understanding these relationships is critical to improving patient engagement, treatment adherence, and overall TB control efforts in this context.

**Aims and objectives:**

To investigate the frequency of depression among TB patients, assess the level of TB stigma, and examine the association between stigma and depression in Districts Peshawar and Haripur.

**Design & methods:**

A cross-sectional study was conducted, from June to December 2023, in Khyber Pakhtunkhwa Province. Non-random convenience sampling was used to recruit 349 participants, of whom 311 were from Peshawar and 38 from Haripur. All participants were evaluated for tuberculosis stigma using the Van Rie TB Stigma Scale. Patients were initially screened for depression via Patient Health Questionnaire-2. A severity assessment was done on 159 depressed participants, using Patient Health Questionnaire-9.

**Results:**

Patient and community perspective stigma were reported as 64.8% and 49.9% in the participants. The frequency of depression among all participants was 45.6%. To evaluate predictors of depression, logistic regression was applied at 5% significance level using STATA 18. In the multivariable analysis, community perspective stigma and residence showed a significant association with depression. However, residence lost its significance when the model was adjusted for patient perspective stigma. Notably, as both patient and community perspective stigma increased, the severity of depression also rose, underscoring the strong association between TB stigma and poor mental health outcomes.

**Conclusions:**

Depression is highly prevalent among tuberculosis patients, and it has a strong relationship with both patient and community perspective stigma. The findings indicate that the higher levels of stigma are associated with greater depression severity while residence shows a context-specific association with mental health outcomes. Integrating stigma reduction and mental health interventions into TB care is essential to improve psychological wellbeing and treatment outcomes among affected individuals.

## Background

TB is a highly stigmatized disease, this is in part due to the contagious nature of tuberculosis combined cultural variations in knowledge, attitudes, and health responses [1]. Patient engagement is essential for effective control of tuberculosis. However, TB stigma, a complex psychosocial phenomenon, is a barrier to engagement with testing and treatment activities [2, 3]. TB stigma is shaped by patient-level factors (e.g., internalized beliefs), community-level influences (e.g., social norms and misconceptions), and health-system-level drivers (e.g., provider attitudes and structural barriers) [4, 5, 12]. Researchers have identified many beliefs that exacerbates a fear of infection leading people to avoid seeking medical assistance and being associated with the disease, which in turn contributes to increasing stigma, both at individual and community levels [4].

Tuberculosis, and its associated stigma contribute to the onset of mental health problems among patients, potentially worsening their overall health status [6, 7]. Tuberculosis infection itself provokes depression because dysregulation of the hypothalamic pituitary-adrenal axis results into emergence of immuno-inflammatory responses and lipid metabolism in the body [8]. Tuberculosis and depression demonstrate a bidirectional relationship, with each condition potentially influencing the onset and progression of the other [7, 11]. Stress stemming from stigma can trigger prolonged autonomic and neuroendocrine reactions, which can elevate the risk of depression by compromising the immune system [8, 9]. Compromised immune function plays a role in both depression and TB, and the relationships between them are exceptionally intricate [10].

This depression among tuberculosis patients leads to decreased adherence to medication [7], voluntary isolation, neglect of self-care routines, and potentially alter the body’s response to Anti-Tuberculosis Treatment (ATT) [8]. TB stigma, collectively contribute to tuberculosis transmission in the community, leading to relapses and fatalities. Despite the recognized burden of tuberculosis-related stigma and depression, there is limited evidence from Pakistan examining their interrelationship, particularly using both patient and community perspectives of stigma. Moreover, context-specific data from Khyber Pakhtunkhwa remain scarce, highlighting the need for this study to better understand how stigma influences mental health outcomes among TB patients in this setting. Investigating and addressing this phenomenon is crucial for achieving optimistic tuberculosis outcomes.

### Objectives:

1. To determine the frequency of depression among patients with tuberculosis in District Peshawar and Haripur.
2. To assess the level of TB stigma among patients with tuberculosis in District Peshawar and Haripur.
3. To examine the association between TB stigma and depression among patients with TB in District Peshawar and Haripur.

## Methods

A cross-sectional study was conducted from June to December 2023. Patients visiting the TB facility and currently on anti-tuberculosis treatments for at least two months in District Peshawar and Haripur of Pakistan were included in the study. We included pulmonary and extra-pulmonary TB patients, however, people with multidrug-resistant TB (MDR-TB) were excluded to avoid the risk of contamination. Other co-morbidities like cancer and HIV were also excluded to prevent the misreporting of the stigma, as these conditions have their own associated stigma.

A sample size of 345 adults aged 18 and over was calculated (using OpenEpi) to provide a 95% confidence interval with a margin of error ± 5%. The calculation assumed a prevalence of depression of 56% in this population [6]. Participants were recruited using non-random convenience sampling, based on the patient distribution reported in the first quarter of 2023 by the Provincial TB Program, until the sample size was achieved.

Initially, data collection was planned at four facilities in Peshawar, including Lady Reading Hospital, District TB Office (DTO) Peshawar (Hashtnagri), Khyber Teaching Hospital, and Government City Hospital, along with two facilities in Haripur—DTO Meelum and District Head Quarter (DHQ) Haripur. We decided to expand the number of facilities, with Gunj and Nahaqi in Peshawar and one Afghan camp in Haripur were added to ensure timely data collection.

In the survey, we employed the Van Rie TB Stigma Scale to evaluate the extent of social stigma experienced by TB patients [7]. This comprised 11 items for community perspective stigma and 12 items for patient perspective stigma. Scores ranged from 0 to 33 for community perspectives and 0 to 36 for patient perspectives. A score of 0 to 11 in the community perspective indicated no stigma, while a score of 12 to 22 was considered low stigma, and a score of 23 to 33 indicated high stigma. On the patient perspective scale, a score of 0 to 12 was considered no stigma, 13 to 24 as low stigma, and 25 to 36 as high stigma. The Van Rie TB stigma scale provides a continuous score without established cut-offs. Therefore, stigma levels were categorized into none, low, and high stigma using tertile-based thresholds to improve interpretability. Categorization of stigma into levels is consistent with broader TB stigma research, where scoring systems are often grouped into categories to aid interpretation [8, 9].

A validated Patient Health Questionnaire-2 (PHQ-2), comprising two questions, with 87% sensitivity and 78% specificity, was utilized for screening depression with ≥ 3 being cut off point [10]. Only depressed participants were assessed with the Patient Health Questionnaire-9 (PHQ-9). This may underestimate or distort severity patterns because PHQ-2 false negatives are not assessed further. The PHQ-9 has a sensitivity of 81.3% and a specificity of 85.3%, was used to gauge the severity of depression [11]. The severity of depression was measured as mild (0–9), moderate [10–14], moderately severe [15–19], and severe [20–27]. Validated Urdu versions PHQ-2 and PHQ-9 were used [12].

Benazir Income Support Program (BISP) poverty scorecard was used to assess the socio-economic status of the patients. It categorises household into six distinct groups based on their score ranges. 0–11 = Extremely/Ultra poor, 12–18 = Chronically poor, 19–23 = transitory poor, 24-34- transitory vulnerable, 35–50 = Transitory non-Poor, 51–100 = non-Poor [13].

The data was analysed using STATA 18, with descriptive statistics used to summarise the demographic characteristics. Inferential statistics were performed to assess associations between dependent and independent variables. Chi-square tests were applied to examine associations between categorical variables, while independent t-tests were used for quantitative variables, age and poverty score. Logistic regression was conducted to estimate the odds ratios for the independent variables, with depression as the single binary outcome variable. A *p*-value threshold of ≤ 0.25 was used for selecting variables for both the univariable and multivariable models, following the purposeful selection approach recommended by Bursac et al. (2008), to ensure that potential confounders that may become significant after adjustment were not excluded prematurely [14]. Variables considered a priori based on existing literature included age, gender, education level, and employment status. Other variables, such as residence (urban/rural) and family structure, were selected empirically if they met the purposeful selection threshold of *p* ≤ 0.25 in the univariable analysis.

Collinearity between patient-perspective and community-perspective stigma was assessed using the Variance Inflation Factor (VIF) and Spearman’s correlation coefficient [15]. A VIF > 5 or a correlation coefficient > 0.7 was considered indicative of significant multicollinearity. Because these two variables were highly correlated we pre-specified that they would be analysed in separate multivariable models to avoid unstable coefficient estimates and to accurately capture the independent effect of each stigma type on depression.

## Results

**Fig. 1:**
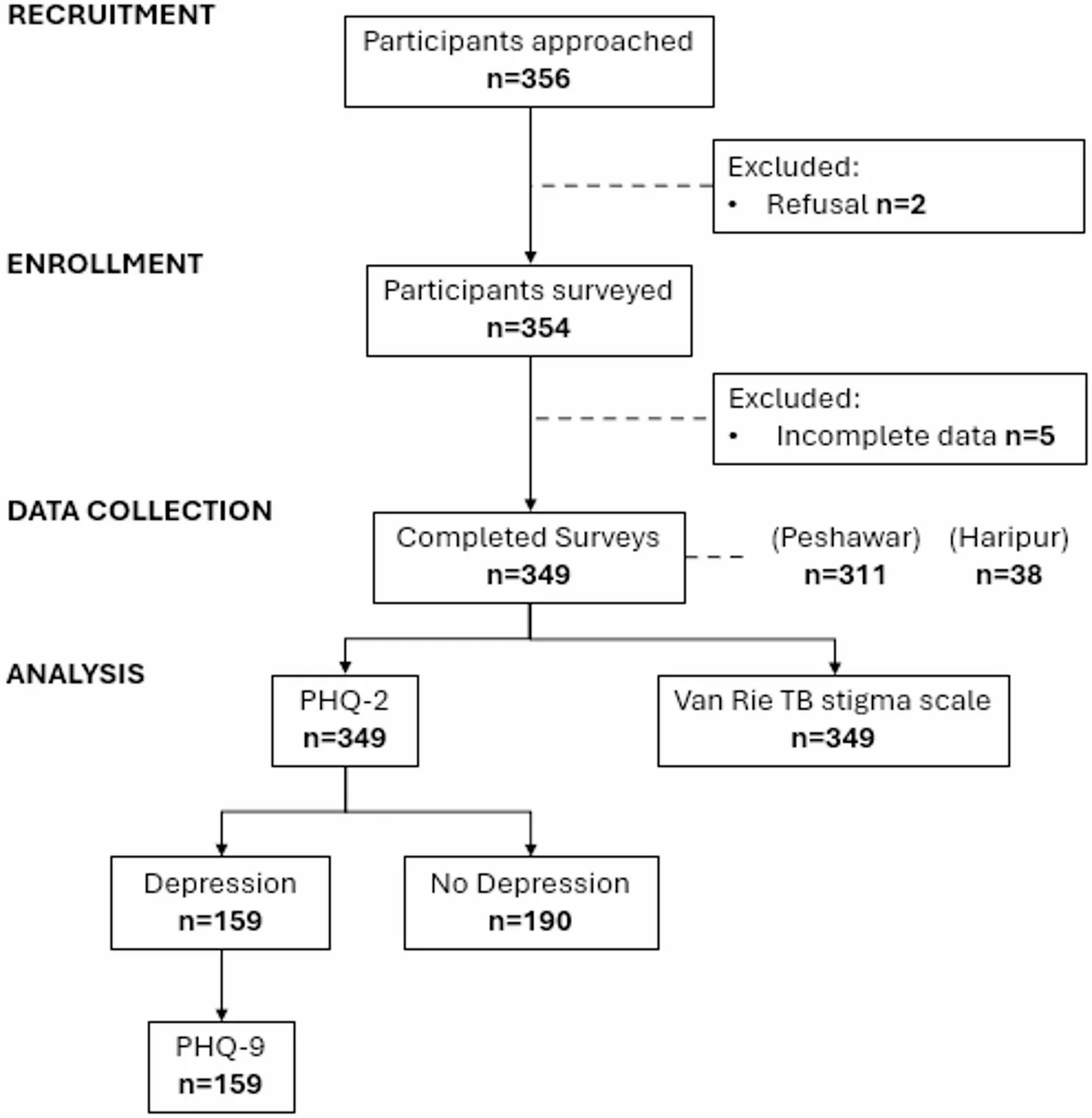
Participant Flow diagram

Data were collected from 349 TB patients, both Afghan and Pakistan nationals, from Peshawar (*n* = 311/349) and Haripur (*n* = 38/349) respectively was considered for analysis (Fig. 1). The disproportionate representation of participants from Peshawar (89%) compared to Haripur (11%) may limit the generalizability of district-level comparisons.

Demographic and clinical details of the study participants and their association with depression status (PHQ-2) is shown in Table 1. There was a slightly higher percentage of females (54.2%), with most participants lived in rural areas (64.2%), and 90.3% were primary/new tuberculosis cases. Pulmonary TB accounted for most cases (72.2%). A notable proportion of participants reported a family history of TB. In total, 159 participants (45.6%) were identified as having depression on the PHQ-2. Only those who were depression positive using the PHQ-2 were assessed for severity of the depression on PHQ-9 (*n* = 159, mean ± SD 14.56 ± 5.78).

**Table 1:** Demographics and Clinical characteristics by depression status (PHQ-2) of all study participants (*n* = 349)

Survey Participants	Depression Status (PHQ-2) *n* (%)		
	Yes 159 (45.6)	No 190 (54.4)	*p*-value
Gender			
Male	72 (45.0)	88 (55.0)	0.847
Female	87 (46.0)	102 (54.0)	
Age			
Mean ± SD (range)	35.98 ± 18.67 (18–90)	32.59 ± 17.11 (18–83)	0.078
Education			
Formal Education*	59 (38.6)	94 (61.4)	0.020
No Formal Education	100 (51.0)	96 (49.0)	
Employment			
Employed~	41 (46.1)	48 (53.9)	0.911
Not employed	118 (45.4)	142 (54.6)	
Nationality			
Pakistani	137 (44.1)	174 (55.9)	0.106
Afghan	22 (57.9)	16 (42.1)	
Residence			
Rural	116 (51.8)	108 (48.2)	0.002
Urban	43 (34.4)	82 (65.6)	
Family Structure			
Joint	120 (47.4)	133 (52.6)	0.254
Nuclear	39 (40.6)	57 (59.4)	
Marital Status			
Unmarried	65 (42.5)	88 (57.5)	0.308
Married	94 (48.0)	102 (52.0)	
Poverty Score (BISP)			
Mean ± SD (range)	55.14 ± 15.83 (24–92)	54.81 ± 15.11 (24–95)	0.093
Tobacco use			
Yes	27 (45.0)	33 (55.0)	0.924
No	132 (45.7)	157 (54.3)	
Type Of TB			
Primary/New	141 (44.8)	174 (55.2)	0.363
Recurrent/Relapse	18 (52.9)	16 (47.1)	
Site Of TB			
Pulmonary	119 (47.2)	133 (52.8)	0.315
Extra-Pulmonary	40 (41.2)	57 (58.8)	
Family History Of TB			
Yes	80 (43.7)	103 (56.3)	0.468
No	79 (47.6)	87 (52.4)	
Chronic Illness			
Yes	40 (51.9)	37 (48.1)	0.202
No	119 (43.8)	153 (56.3)	
Month of treatment			
2–4 Months	110 (47.4)	122 (52.6)	0.524
> 4–6 Months	41 (43.2)	54 (56.8)	
> 6 Months	8 (36.4)	14 (63.6)	

* Formal Education  Primary, Secondary, Intermediate, and Tertiary Education

*~ Employed* **=** Government, and Private

Rural residents showed higher rates of depression than urban residents (51.8% vs. 34.4%, *p* = 0.002), while participants with no formal education were more likely to be depressed compared to those with formal education (51.0% vs. 38.6%, *p* = 0.020). There was a statistically significant association between stigma and depression, with a higher proportion of participants screening positive for depression observed in higher stigma categories. Other demographic variables, including gender, marital status, employment, TB type, and treatment duration, showed no significant association with depression (*p* > 0.05). Other demographic variables, including gender, marital status, employment, TB type, and treatment duration, showed no significant association with depression (*p* > 0.05).

Participants with higher levels of both patient and community perspective stigma showed markedly higher rates of depression (Table 2). There is a statistically significant association between patient perspective stigma and depression severity (*p* = 0.001) (Table 3).

**Table 2:** Stigma status of all participants (*n* = 349) by Depression Status (PHQ-2)

Stigma Status	Depression Status (PHQ-2) *n* (%)		
	Yes 159 (45.6)	No 190 (54.4)	*p*-value
Patient Perspective			
High (*n* = 54)	50 (92.6)	4 (7.4)	0.001
Low (*n* = 120)	61 (50.8)	59 (49.2)	
None (*n* = 175)	48 (27.4)	127 (72.6)	
Community Perspective			
High (*n* = 86)	66 (76.7)	20 (23.3)	0.001
Low (*n* = 140)	57 (40.7)	83 (59.3)	
None (*n* = 123)	36 (29.3)	87 (70.7)	

**Table 3:** Stigma Status of depressed participants (*n* = 159) by severity of depression (PHQ-9)

Stigma Status	Severity of Depression (PHQ-9) *n* (%)				
	Mild Depression	Moderate Depression	Moderately Severe Depression	Severe Depression	*p*-value
Patient Perspective					
High (*n* = 50)	2 (4.0%)	6 (12.0%)	25 (50.0%)	17 (34.0%)	0.001
Low (*n* = 61)	19 (31.1%)	13 (21.3%)	15 (24.6%)	14 (23.0%)	
None (*n* = 48)	16 (33.3%)	16 (33.3%)	12 (25.0%)	4 (8.4%)	
Community Perspective					
High (*n* = 66)	8 (12.1%)	9 (13.6%)	30 (45.5%)	19 (28.8%)	0.004
Low (*n* = 57)	20 (35.1%)	16 (28.1%)	11 (19.3%)	10 (17.5%)	
None (*n* = 36)	9 (25.0%)	10 (27.8%)	11 (30.5%)	6 (16.7%)	

## Logistic regression model for depression and TB stigma

Those with high patient perspective stigma had the highest depression prevalence (92.6%), compared to much lower rates among low or no-stigma groups. A similar trend was observed for community perspective stigma, with depression most common among those reporting high community stigma (76.7%). In both stigma scales, depression decreased progressively as stigma levels declined. These associations were statistically significant (*p* = 0.001), indicating that stigma is strongly linked with depressive symptoms among TB patients. We collected data on the severity of depression (using PHQ-9) for the 159 participants with depression (according to PHQ-2).

The univariable logistic regression analysis examined sociodemographic and stigma related factors for their association with depression (using PHQ-2) among 349 survey participants (Table 4). The following variables were significantly associated with depression. Participants with formal education had 40% lower odds of depression compared with those without formal education (OR = 0.60, 95% CI: 0.39–0.92, *p* = 0.021). Participants living in urban areas had lower odds of depression compared with those in rural areas (OR = 0.48, 95% CI: 0.31–0.76, *p* = 0.002), indicating an association between residence and depression. Regarding socioeconomic status, individuals classified as transitory non-Poor had significantly reduced odds of depression (OR = 0.45, *p* = 0.049), whereas those classified as non-Poor did not show a significant association (*p* = 0.297).

**Table 4:** Univariable analysis of the potential predictors of depression among all TB patients (*n* = 349)

Survey Participants	Depression Status (PHQ-2)	
	OR (95%CI)	*p*-value
Age		
Older Adults (60 above)	1.43 (0.80–2.55)	0.215
Middle aged Adults (40–59)	1.45 (0.79-2.64)	0.225
Younger Adults (18–39)	REF	
Education		
Formal Education	0.60 (0.39-0.92)	0.021
No Formal Education	REF	
Residence		
Urban	0.48 (0.31- 0.76)	0.002
Rural	REF	
Nationality		
Afghan	1.74(0.88-3.45)	0.109
Pakistani	REF	
Poverty Score (BISP)		
Transitory non-Poor	0.45(0.21- 0.99)	0.049
non-Poor	0.68(0.33 − 1.40)	0.297
Transitory Vulnerable	REF	
Chronic Illness		
Yes	1.38 (0.83 − 2.30)	0.203
No	REF	
Family Structure		
Nuclear	0.75(0.47-1.22)	0.255
Joint	REF	
Stigma Status		
Patient Perspective		
Present	4.66(2.96–7.33)	0.001
Absent	REF	
Severity Of Stigma		
High	33.07(11.33–96.53)	0.001
Low	2.72(1.67–4.45)	0.001
None	REF	
Community Perspective		
Present	2.88 (1.80–4.61)	0.001
Absent	REF	
Severity Of Stigma		
High	7.97 (4.23–15.02)	0.001
Low	1.65 (0.99–2.77)	0.054
None	REF	

Stigma related variables showed the strongest associations with depression. Participants reporting patient perspective stigma had five times higher odds of depression (OR = 4.66, 95% CI: 2.96–7.33, *p* = 0.001). Higher levels of patient-perceived stigma were associated with markedly increased odds of depression (OR = 33.07, 95% CI: 11.33–96.53, *p* = 0.001), and low stigma also significantly increasing the odds (OR = 2.72, *p* = 0.001). Similarly, community perspective stigma was significantly associated with depression; participants reporting stigma from the community had almost three times higher odds of depression than those reporting no stigma (OR = 2.88, 95% CI: 1.80–4.61, *p* = 0.001). Severity analysis further supported this association, as high community perspective stigma increased the odds of depression (OR = 7.97, 95% CI: 4.23–15.02, *p* = 0.001), whereas low stigma showed a borderline, non-significant association (OR = 1.65, *p* = 0.054).

Due to collinearity between patient and community perspective stigma, we kept both the variables separately in the model and then performed multivariable logistic regression (Table 5).

**Table 5:** Multivariable Analysis of the potential predictors of depression among TB Patients among TB patients by patient‑perceived and community‑perceived stigma (*n* = 349)

Survey Participants	Depression Status (PHQ-2)			
	Patient Perspective Stigma		Community Perspective Stigma	
	OR (95%CI)	*p*-value	OR (95%CI)	*p*-value
Age				
Older Adults (60 above)	1.74 (0.81– 3.73)	0.153	1.21 (0.58–2.51)	0.604
Middle aged adults (40–59)	1.69 (0.80–3.54)	0.167	1.22 (0.60–2.49)	0.577
Younger Adults (18–39)	REF		REF	
Education				
Formal Education	0.76 (0.45–1.29)	0.318	0.73 (0.44–1.19)	0.207
No Formal Education	REF		REF	
Residence				
Urban	0.63 (0.37–1.07)	0.087	0.56 (0.34–0.92)	0.022
Rural	REF		REF	
Poverty Score (BISP)				
Transitory non-Poor	0.56 (0.23–1.35)	0.196	0.56 (0.24–1.31)	0.181
non-Poor	0.79 (0.34–1.83)	0.581	0.90 (0.40–2.02)	0.801
Transitory Vulnerable	REF		REF	
Nationality				
Afghan	1.40 (0.63–3.10)	0.410	1.46 (0.67–3.16)	0.338
Pakistani	REF		REF	
Chronic Illness				
Yes	1.09 (0.56–2.14)	0.792	0.97 (0.51–1.84)	0.930
No	REF		REF	
Family Structure				
Nuclear	0.72 (0.41–1.27)	0.261	0.77 (0.45–1.32)	0.343
Joint	REF		REF	
Stigma				
High	31.05 (10.42–92.48)	0.001	6.94 (3.63–13.28)	0.001
Low	3.00 (1.78–5.06)	0.001	1.69 (0.99–2.87)	0.055
None	REF		REF	

## Logistic model for patient perspective stigma

All severity analyses are restricted to the 159 participants who screened positive on PHQ-2 and were then assessed using PHQ-9. High stigma is strongly associated with more severe forms of depression. Only 5.4% of individuals with high stigma reported mild depression, while 48.1% had moderately severe depression and 48.6% had severe depression. Low stigma shows a more even distribution, but still with a considerable proportion experiencing moderate to severe depression. No stigma is associated with lower levels of depression severity. A larger proportion of participants with no stigma fall in the mild (43.3%) and moderate (45.7%) categories, and only 11.4% in the severe category. Overall, as patient perspective stigma increases the severity of depression also increases, reinforcing the strong negative psychological impact of TB stigma.

After adjusting for age, education, residence, poverty status, nationality, chronic illness, and family structure, patient perspective stigma remained the strongest predictor of depression. Participants experiencing elevated levels of patient perspective stigma had significantly increased odds of depression (OR = 31.05, 95% CI: 10.42–92.48, *p* = 0.001). Participants reporting low stigma had higher odds of depression compared with the no-stigma group (OR = 3.00, *p* = 0.001), indicating a positive association between stigma level and depression.

None of the other variables in this model remained statistically significant after adjustment. Age groups, education status, poverty score, nationality, chronic illness, and family structure did not show independent associations with depression. Residence showed a borderline association, with urban participants showing lower odds of depression (OR = 0.63, *p* = 0.087), but this did not reach statistical significance. Overall, the results indicate that patient perspective stigma is the dominant and independent predictor of depression in this model.

## Logistic model for community perspective stigma

There is also a significant association between community perspective stigma and depression severity (*p* = 0.004) (Table 3). High community perspective stigma is related to higher rates of severe and moderately severe depression. For example, 57.7% of individuals with high stigma reported moderately severe depression, and 54.3% reported severe depression. Low stigma shows a mixed distribution, with fewer participants in the severe category. No stigma is more frequently associated with milder forms of depression. For instance, 24.3% had mild depression, and only 17.1% had severe depression. Higher community perspective stigma is associated with greater depression severity.

In the adjusted model examining community perspective stigma, strong associations with depression also persisted. Participants reporting high community stigma had significantly higher odds of depression (OR = 6.94, 95% CI: 3.63–13.28, *p* = 0.001). Low community stigma showed a borderline but non-significant association (OR = 1.69, *p* = 0.055), suggesting a trend but not a confirmed effect after adjustment.

Among the other variables, urban residence remained significant, with urban residents having lower odds of depression compared with rural participants (OR = 0.56, 95% CI: 0.34–0.92, *p* = 0.022). All other variables including age, education, poverty status, nationality, chronic illness, and family structure, did not show statistically significant associations after adjustment.

These findings indicate that both patient and community perspective stigma are strong predictors of depression. Additionally, living in an urban area appears to offer a modest protective effect. Overall, the results highlight the critical role of TB stigma in influencing the mental health of TB patients, underscoring the need for targeted psychosocial interventions to reduce stigma and its psychological consequences.

## Discussion

This cross-sectional study investigated the prevalence of depression and its association with stigma among patients with tuberculosis (TB). Both patient and community perspective stigma were found to have significant association with depression. In the community perspective model, residence was also significantly associated with depression, indicating the role of contextual and environmental factors in shaping the mental health of TB patients. The frequency of depression among TB patients in this study was 45.6% (males: 45%, females: 46%), with no marked gender difference. This prevalence closely aligns with the pooled estimates reported by Duko et al. (2020), who found rates of 45.25% among males and 51.54% among females [16]. However, it is slightly lower than the rates reported in previous studies conducted in various regions of Pakistan, including Karachi (56%), Peshawar (65.5%), Faisalabad (70–80%), Lahore (62%), and Rawalpindi (42.5%) [6, 17–20]. These discrepancies may be attributed to differences in study design, TB type (e.g., Multidrug-resistant TB (MDR-TB) versus pulmonary or extrapulmonary TB), sample characteristics, and the use of different assessment tools. Despite these variations, the consistently high prevalence highlights the persistent challenge of depression among TB patients across regions in Pakistan. Most participants in this study were young adults with lower levels of education, like findings reported by other Pakistani studies [21–23].

Interestingly, the majority of our participants were categorized as non-Poor according to the BISP poverty score, which differs from previous research linking TB with lower socioeconomic status [22, 24, 25]. This should not be interpreted as indicating that TB is unrelated to poverty. The discrepancy may reflect differences in how financial well-being was assessed, while other studies directly inquired about income. Our study used the BISP poverty score as a proxy measure of socioeconomic status, which may classify participants differently.

In the community perspective model, urban residents had a lower likelihood of depression compared to rural residents, consistent with Elavarasi et al. (2024) [26]. This difference could possibly be explained by factors such as greater awareness, education, and access to healthcare services in urban populations. In contrast, patients in rural areas often face logistical barriers such as limited transportation options and fixed clinic hours, which can exacerbate emotional distress and hinder access to timely treatment. Therefore, these findings should be interpreted cautiously, as they do not provide definitive evidence of a substantive urban–rural difference.

Our study also revealed that higher patient-perceived stigma was associated with increased odds of depression TB compared to those who did not. Similar findings were reported in other studies, who identified internalized stigma as a significant predictor of depressive symptoms among TB patients [24, 27]. The experience of stigma may lead to feelings of shame, social withdrawal, and hopelessness, thereby increasing vulnerability to depression. Additionally, consistent with recent findings [28], residence is significantly associated with depression in our study, underscoring the influence of place-based disparities on mental health outcomes.

In our study, the very high odds ratio observed for the high patient-perceived stigma category (OR = 33.07) should be interpreted with caution. This extreme estimate likely reflects sparse-data bias due to small cell counts in the subgroup with both high stigma and depression, as suggested by the wide confidence interval. While the association remains statistically significant and clinically relevant, the precise magnitude of the OR should be interpreted as an indicator of a very strong association rather than a point-exact estimate.

In contrast, other factors such as age, education, and socioeconomic status were not significantly associated with depression in our analysis. This differs from the findings of several recent studies, who identified female gender, low socioeconomic status, poor social support, and comorbidities as significant predictors of depression among TB patients [16, 28–30]. These inconsistencies may reflect contextual differences, varying measurement tools, or sample characteristics. They also suggest that psychosocial variables—particularly stigma—may play a more dominant role in determining mental health outcomes than demographic factors alone.

Our study builds upon the dual-perspective approach proposed by Van Rie et al. (2008), demonstrating that both internalized (patient perspective) and enacted (community perspective) stigma substantially contribute to depression among TB patients [7]. Examining both types of stigma offers a more complete picture of the barriers affecting TB care. This broader understanding can help programme designers develop stigma-reduction strategies that address both individual patient experiences and community attitudes, thereby improving engagement with TB services. Similar findings from South Korea [24] and Nepal [27] reinforce that addressing stigma at both personal and community levels is essential for effective mental health support in TB care.

Our findings have important public health implications for TB programmes. Routine screening for depression and stigma should be incorporated into TB services, with prompt referral pathways for counselling or mental health care when needed, because WHO recommends integrating mental health care into TB services and training staff to avoid stigmatizing practices [31]. In parallel, stigma-reduction interventions such as patient education, peer or psychosocial support, and health worker training should be embedded within programme design to improve engagement, adherence, and overall TB treatment outcomes [32].

### Limitations

The study has several important limitations. First, there is a possibility of potential underestimation of severe depression if PHQ-2 false negatives occurred, because PHQ-2 was used to screen all participants, whereas PHQ-9 was administered only to those who screened positive.

Second, although HIV and cancer patients were excluded to avoid potential misreporting of stigma, these exclusions reduce the generalizability of the findings. Additionally, key confounders such as social support, treatment adherence, multidrug-resistant TB status, and other comorbidities were not measured, limiting the ability to fully account for factors influencing depression and stigma. The representativeness of the sample is also a concern, as nearly 90% of participants were recruited from a single district (Peshawar), restricting the applicability of results to the broader Khyber Pakhtunkhwa population. Finally, the analysis does not account for treatment adherence, which is an important determinant of both stigma and mental health outcomes. Another limitation of this survey is the risk of response bias, as some participants may have been uncomfortable answering sensitive questions in the presence of others, and many were unaware of the HIV/AIDS–TB link included in the Van Rie TB Stigma Scale (Van Rie 2008), leading to confusion and potentially inaccurate or underestimated stigma-related responses. Although interviews were conducted in designated areas and interviewers aimed to ensure privacy, complete confidentiality could not always be guaranteed in busy clinic environments, meaning that some participants may have answered sensitive questions in the presence or near-presence of others, potentially introducing response bias. In addition, we tried to ensure the Van Rie TB Stigma Scale was locally and culturally appropriate [8]. We used simple forward and back translation by bilingual team members. However, we did not undertake a validation of the translated the Van Rie TB Stigma Scale. This may further limit the contextual validity and interpretability of the stigma scores. Cross-sectional design of the study is also a limitation hence Causality cannot be inferred; reverse causation (depression increasing perceived stigma) is possible. Some additional facilities were added during fieldwork “to ensure timely data collection, this may have implications for selection bias.

Survey did facility-based recruitment and there is possibility that patients disengaged from treatment, who may be more stigmatised or depressed, were not represented. Because recruitment was facility based and restricted to patients currently attending TB services, individuals who had discontinued or disengaged from treatment were not represented in our sample; this may have led to underestimation of stigma and depressive symptoms, as those who drop out of care are often more vulnerable and may face greater psychosocial and structural barriers.

## Conclusion

This study demonstrates that both patient and community perspective stigma were strongly associated with depressive symptoms, while the impact on treatment adherence is plausible but was not directly measured here. Higher levels of stigma were strongly associated with increased depression severity, highlighting the profound psychological burden experienced by TB patients. In addition to treating depression in tuberculosis patients, it is important to introduce interventions to reduce stigma.

Implementing measures to address and mitigate stigma and depression can play a crucial role in improving the overall well-being and treatment outcomes for individuals affected by tuberculosis. These findings emphasize the urgent need to integrate stigma reduction strategies or stigma-sensitive health education and mental health support into TB control programs.

Interventions should target both individual and community levels to address misconceptions, promote social acceptance, and provide psychological care for affected individuals. Addressing stigma holistically is essential not only for improving mental health outcomes but also for enhancing treatment adherence and the overall success of TB control efforts.

## Data Availability

The datasets used and/or analysed during the current study are available from the corresponding author on reasonable request.

## Abbreviations

ATT: Anti–Tuberculosis Treatment
BISP: Benazir Income Support Program
DTO: District TB Office
DHQ: District Headquarters
MDR: TB–Multidrug–resistant TB
PHQ: 2–Patient Health Questionnaire–2
PHQ: 9–Patient Health Questionnaire–9
TB: Tuberculosis
